# Bullosis diabeticorum: A distinctive blistering eruption in diabetes mellitus

**DOI:** 10.4103/0973-3930.50714

**Published:** 2009

**Authors:** Sudip Kumar Ghosh, Debabrata Bandyopadhyay, Gobinda Chatterjee

**Affiliations:** Department of Dermatology, Venereology, and Leprosy, R.G. Kar Medical College; 1, Khudiram Bose Sarani, Kolkata- 700 004, W.B, India

**Figure 1:**
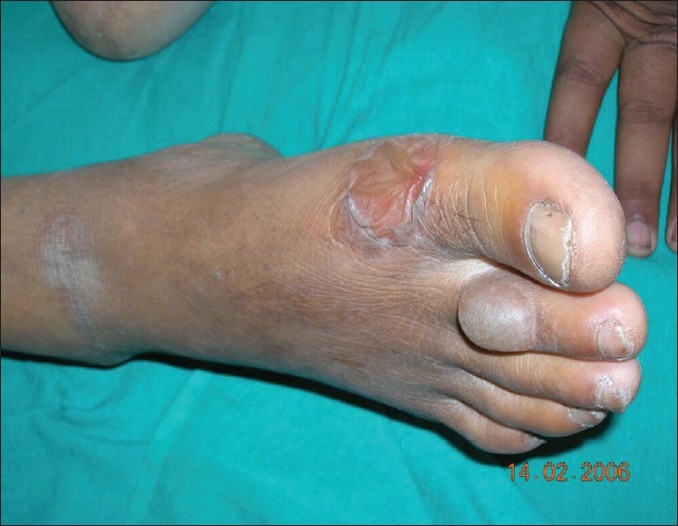
Tense bulla on a nonerythematous base on the dorsum of the second toe of the right toe and a larger collapsed bulla on the ball of the great toe

Dear Sir,

A 41- year-old man with known type-1 diabetes presented with acute onset, asymptomatic, spontaneous, tense blisters of two days' duration on his right foot. The patient was on human insulin and there was no history of trauma or friction from footwear prior to the eruption. There was no history of photosensitivity and the patient could not recall any new drug intake in the preceding couple of weeks. He used to maintain meticulous foot hygiene, according to his physician's instructions. On examination, a tense, nontender blister on a nonerythematous base was seen on the dorsum of the second toe of the right foot. A larger collapsed bulla was also present on the ball of the great toe [Figure 1]. Histopathology of the lesional skin showed a subepidermal bulla without any inflammatory infiltrate. A direct immunofluorescence test was negative, thus excluding any immunobullous disease. No specific treatment was offered to the patient for the bullae. Within three weeks, the patient recovered uneventfully with slight residual dyspigmentation, but without any scarring. Based on the clinical, histopathological, and immunofluorescence pattern, the patient was diagnosed to have bullosis diabeticorum.

Bullosis diabeticorum, also known as bullous disease of diabetes and diabetic bullae, is a rare, distinct, spontaneous, noninflammatory, blistering condition of unknown etiology occurring in the setting of diabetes mellitus.[1] While Cantwell, and Martz named the condition in 1967, Krane first reported this condition in 1930. The exact etiology of bullosis diabeticorum is not known but it is thought to be multifactorial in origin. We could not find any reference in the existing literature about the relationship of the occurrence of diabetic bulla and the degree of metabolic derangement or glycemic control. It has been reported to involve approximately 0.5% of diabetic patients of the US population[2] and one recent Indian study showed involvement of 2% of the diabetic population.[3] There is a male preponderance of the disease with a male-to-female ratio of 2:1 and the age range varies from 17 to 84 years.[2]

The blisters have a propensity to be large and often have an asymmetrical shape. Although they are much more common over the acral areas and are more common on the lower extremities, nonacral sites (*e.g*., the trunk) may also be involved. Other differential diagnoses to be considered in these cases are friction blisters, bullous fixed drug reactions, bullous pemphigoid, bullous SLE, and epidermolysis bullosa acquisita. Routine histopathological investigations of diabetic bullae show nonspecific features including an intraepidermal or subepidermal bulla; the inflammatory element is absent or trivial.[2] Direct immunofluorescence reveals no primary immunological abnormality and hence, is noncontributory.[2] Monitoring for secondary infection and differentiation from other blistering dermatoses are very important. The characteristic histopathological features of frictional blisters (intraepidermal necrosis), bullous fixed drug reaction (basal cell degeneration, inflammatory infiltrates), bullous pemphigoid (inflammatory infiltrates), bullous SLE (basal cell degeneration, inflammatory infiltrates), and epidermolysis bullosa acquisita (inflammatory infiltrates) are conspicuous by their absence in diabetic bullae, as in the present case. Furthermore, a negative DIF test may reasonably exclude the possibilities of bullous pemphigoid, bullous SLE, and epidermolysis bullosa acquisita. There is no specific treatment of the condition and the bullae usually heal spontaneously without scarring.[4] Although the disease often recurs and may be complicated with secondary infection or rarely by osteomyelitis, the overall prognosis is usually excellent.
